# Prognostic value and risk stratification of residual disease in patients with incidental gallbladder cancer

**DOI:** 10.1186/s12957-020-1794-2

**Published:** 2020-01-24

**Authors:** Emilio Ramos, Nuria Lluis, Laura Llado, Jaume Torras, Juli Busquets, Antoni Rafecas, Teresa Serrano, Kristel Mils, David Leiva, Joan Fabregat

**Affiliations:** 1 Department of Surgery, IDIBELL, Hospital Universitario de Bellvitge, CIBERehd, Servicio de Cirugía General y Digestiva, Universidad de Barcelona, Av Feixa Llarga s/n, 08907 L’Hospitalet de Llobregat, Barcelona Spain; 2 Department of Pathology, Hospital Universitario de Bellvitge, L’Hospitalet de Llobregat, Spain; 3 Department of Radiology, Hospital Universitario de Bellvitge, L’Hospitalet de Llobregat, Spain

**Keywords:** Hepatobiliary neoplasms, Incidental gallbladder Cancer, Risk score, Residual disease, Staging, Surgical treatment

## Abstract

**Background and aim:**

Given their poor prognosis, patients with residual disease (RD) in the re-resection specimen of an incidental gallbladder carcinoma (IGBC) could benefit from a better selection for surgical treatment. The Gallbladder Cancer Risk Score (GBRS) has been proposed to preoperatively identify RD risk more precisely than T-stage alone. The aim of this study was to assess the prognostic value of RD and to validate the GBRS in a retrospective series of patients.

**Material and methods:**

A prospectively collected database including 59 patients with IGBC diagnosed from December 1996 to November 2015 was retrospectively analyzed. Three locations of RD were established: local, regional, and distant. The effect of RD on overall survival (OS) was analyzed with the Kaplan-Meier method. To identify variables associated with the presence of RD, characteristics of patients with and without RD were compared using Fisher’s exact test. The relative risk of RD associated with clinical and pathologic factors was studied with a univariate logistic regression analysis.

**Results:**

RD was found in 30 patients (50.8%). The presence of RD in any location was associated with worse OS (29% vs. 74.2%, *p* = 0.0001), even after an R0 resection (37.7% vs 74.2%, *p* = 0.003). There was no significant difference in survival between patients without RD and with local RD (74.2% vs 64.3%, *p* = 0.266), nor between patients with regional RD and distant RD (16.1% vs 20%, *p* = 0.411). After selecting patients in which R0 resection was achieved (*n* = 44), 5-year survival rate for patients without RD, local RD, and regional RD was, respectively, 74.2%, 75%, and 13.9% (*p* = 0.0001). The GBRS could be calculated in 25 cases (42.3%), and its usefulness to predict the presence of regional or distant RD (RDRD) was confirmed (80% in high-risk patients and 30% in intermediate risk *p* = 0.041).

**Conclusion:**

RDRD, but not local RD, represents a negative prognostic factor of OS. The GBRS was useful to preoperatively identify patients with high risk of RDRD. An R0 resection did not improve OS of patients with regional RD.

## Background

Since the widespread adoption of the laparoscopic approach, the number of patients diagnosed with incidental gallbladder carcinoma (IGBC) has increased. Although there is some controversy, the most common definition of IGBC is a histologic diagnosis of malignancy after an elective cholecystectomy for presumed benign disease. This is the definition used in the present study.

Re-resection of patients with IGBC is recommended in T1b, T2, and T3 tumors without disseminated disease. The main goal of the reoperation is to resect the possible residual disease (RD), in order to improve patient’s survival and to perform a correct staging [1–8].

The prognostic value of RD has received much attention in recent years [3–5, 7–11] and it has been suggested that surgery does not improve survival of patients with RD [11–13]. Patients with known or high risk of RD might benefit from a specific strategy that could include an extended staging study, a longer observation period before re-resection, the administration of preoperative chemotherapy (CHT), and an exploratory laparoscopy before the reoperation [7]. This strategy could improve patient selection prior to attempt re-resection.

Given the limitations of imaging studies for preoperative staging [12, 14, 15], attempts have been made to predict RD risk from the pathological data of the cholecystectomy specimen. In this sense, the Gallbladder Cancer Predictive Risk Score (GBRS) [16] has been proposed but not yet validated. The aim of the present study was to assess the prognostic value of RD and to validate the GBRS in a retrospective series of patients with IGBC.

## Patients and method

The Gallbladder Cancer Predictive Risk Score (GBRS) published in 2016 [16] was developed using T-stage, tumor grade, presence of lymphovascular invasion (LVI) and perineural invasion (PNI) determined in the cholecystectomy specimen. Each factor was assigned a value which was added to obtain a total risk score ranging from 3 to 10. The scores were then separated into three risk groups: low (3–4), intermediate (5–7), and high (8–10). The primary endpoint of the authors was to assess the predictive value of the GBRS in finding loco-regional or distant RD at the time of re-resection for IGBC.

From December 1996 to November 2015, data from 60 consecutive patients diagnosed with IGBC who underwent a re-resection was obtained from a prospectively maintained database. Before the reoperation, patients underwent a physical examination, and blood samples were obtained. Imaging techniques included an enhanced thoraco-abdominal CT scan in all cases and, since 2010, a PET/CT FDG scan and an MRI. Reports and histological preparations of cholecystectomy specimen performed in outside hospitals were reviewed by an expert pathologist from our center. Radical resection was recommended in all patients who presented stage T1b or higher, or cystic margin invasion without evidence of disseminated disease.

Surgery consisted of a resection of the vesicular bed or an anatomical resection of liver segments IVb/V. A lymphadenectomy of the hepatic hilum was associated in all cases. Bile duct resection was carried out in patients with cystic margin involvement and in two patients in whom common bile duct ischemia after lymphadenectomy was suspected.

Surgical excision of the port sites was only carried out in selected cases. Postoperative mortality was defined by death within the first 90 days after surgery.

The presence of RD was established by the pathology findings in intraoperative samples or in the resected specimen. Three locations of RD were established: (1) local, when isolated non-discontinuous involvement of the vesicular bed or the cystic stump was found; (2) regional, which included common bile duct involvement, PNI, LVI, lymph node invasion, or invasion of neighboring organs; and (3) distant, when discontinuous hepatic involvement (metastasis) or peritoneal carcinomatosis were present.

Staging after re-resection was obtained with data from both surgeries according to the 7th edition of the TNM classification of the American Joint Committee on Cancer (AJCC). Follow-up included a physical examination, blood tests, and a thoraco-abdominal CT scan every 6 months. Recurrences were confirmed by histologic or radiological findings. Patients’ data were anonymized, and the study protocol was approved by the Bellvitge University Hospital’s Clinical Research Ethic Committee (PR072/18).

### Statistical analysis

Results are presented as proportions for qualitative variables and as mean and standard deviation for continuous variables. Survival was calculated from the reoperation date until death or end of follow-up.

In order to determine the prognostic value of RD on OS, survival curves were constructed using the Kaplan-Meier method and compared with the log-rank test. To identify variables associated with the presence of RD, the characteristics of patients with and without RD were compared using the Fisher’s exact test. Statistical significance was considered when *p* < 0.05. To assess the relative risk (RR) of RD associated with clinical and pathologic factors, univariate logistic regression analysis was performed.

As the tumor was usually diagnosed at outside institutions, potentially relevant pathology information was missing for many patients. To determine if the samples with incomplete information were biased, the analysis of prognostic factors included unknown as a separate category. Analysis was performed using IBM SPSS Statistics 25.0 (IBM Ins, Armonk, NY).

## Results

A patient with T*is* stage in the cholecystectomy specimen was excluded from the study due to a diffuse involvement of the entire biliary tract epithelium identified in the reoperation. The demographic characteristics of the remaining 59 patients and the pathology findings of the cholecystectomy specimen are presented in Table 1. No patient received preoperative chemotherapy. RD was found in 30 patients (50.8%). Distant (*n* = 9) or extensive regional disease (*n* = 1), not detected in preoperative staging, was found in 10 patients at reoperation and thus resection was performed in 49 patients (44 R0 and 5 R1). No patient died in the postoperative period.

**Table 1:** Demographic characteristics of the complete series, pathologic data from the cholecystectomy specimen, and operative data at re-resection. Comparison with the Ethun et al. series [16]

		Ramos et al., *n* = 59	Ethun et al., *n* = 262	*P* (unknown excluded)
Age (years), mean (SD)		65 (9.6)	65 (11.6)	1
Sex, M/F		18/41		
ASA, I/II/III/IV		17/32/9/1		
Cholecystectomy, urgent/elective		15/44		
Surgical approach, open or converted/laparoscopic		19/40		
T-stage, *n* (%)	Unknown	–	36 (13.7)	< 0.001
	T(1a + 1b)	5 (8.5)	22 (8.3)	
	T2	46 (78)	113 (43)	
	T3/T4	8 (13.6)	91 (34.7)	
*N*, N0/N1/unknown		11/15/33		
Affected cystic margin, Y/N/unknown		16/30/13		
Grade of differentiation *n* (%)	Unknown	17 (29)	67 (25.5)	0.040
	Well	11 (18.3)	24 (9.1)	
	Moderately	24 (40)	115 43.8)	
	Poorly	7 (11.7)	56 (21.3)	
Histology, adenocarcinoma/other		48/11		
Lymphovascular invasion, *n* (%)	Unknown	12 (20.3)	149 (57)	0.136
	No	32 (54.2)	61 (23.2)	
	Yes	15 (5.4)	52 (19.8)	
Perineural invasion, *n* (%)	Unknown	24 (40.7)	145 (55.3)	
	No	19 (32.2)	55 (21)	0.369
	Yes	16 (27.1)	62 (23.6)	
GBRS	Unknown	34/59	174/262	
	High	5 (20)	42 (48)	0.030
	Intermediate	20 (80)	42 (48)	
	Low	0	4 (4)	
Time to reoperation (weeks), mean (SD)		12.4 (6.2)	9.3 (14.3)	0.010
Completed re-resection, *n* (%)		49 (83)	214 (82)	0.804
Liver resection, *n* (%)		46/49 (94)	191 (89)	0.327
Common bile duct resection, *n* (%)		23/49 (47)	73 (34)	0.092
Port-site excision, *n* (%)		16/49 (32)	87 (40)	0.677
Final margin status R0, *n* (%)		44 (74.5)	196 (75)	0.974
RD at reoperation, *n* (%)		30 (50.8)	174 (66.4)	0.05
Local or regional		21 (35.5)	129 (49.2)	
Distant		9 (15.3)	45 (17.2)	
Final TNM staging, 7th ed.	0,I/II/IIIa,IIIb,IV	5/22/32		

The characteristics of our patients are compared with those of Ethun et al. [16] in Table 1. The latter have a greater proportion of unknown data, a more advanced tumor stage, and a higher incidence of RD. No differences were seen in aspects related to the surgical technique, except for the time interval between both operations, which was longer in our series.

### Prognostic value of RD and influence of its anatomical location

Patients with confirmed RD at reoperation had a lower 5-year survival rate than patients without RD (29% vs. 74.2%, *p* = 0.0001) even after an R0 resection (37.7% vs 74.2%, *p* = 0.003).

The 5-year survival rate of patients with local RD, regional RD, and distant RD was 64.3%, 16.1%, and 20%, respectively. Patients without RD and patients with local RD had similar 5-year survival rate (*p* = 0.266). The 5-year survival rate of patients with regional RD was similar to that of patients with distant RD (*p* = 0.411).

**Fig. 1:**
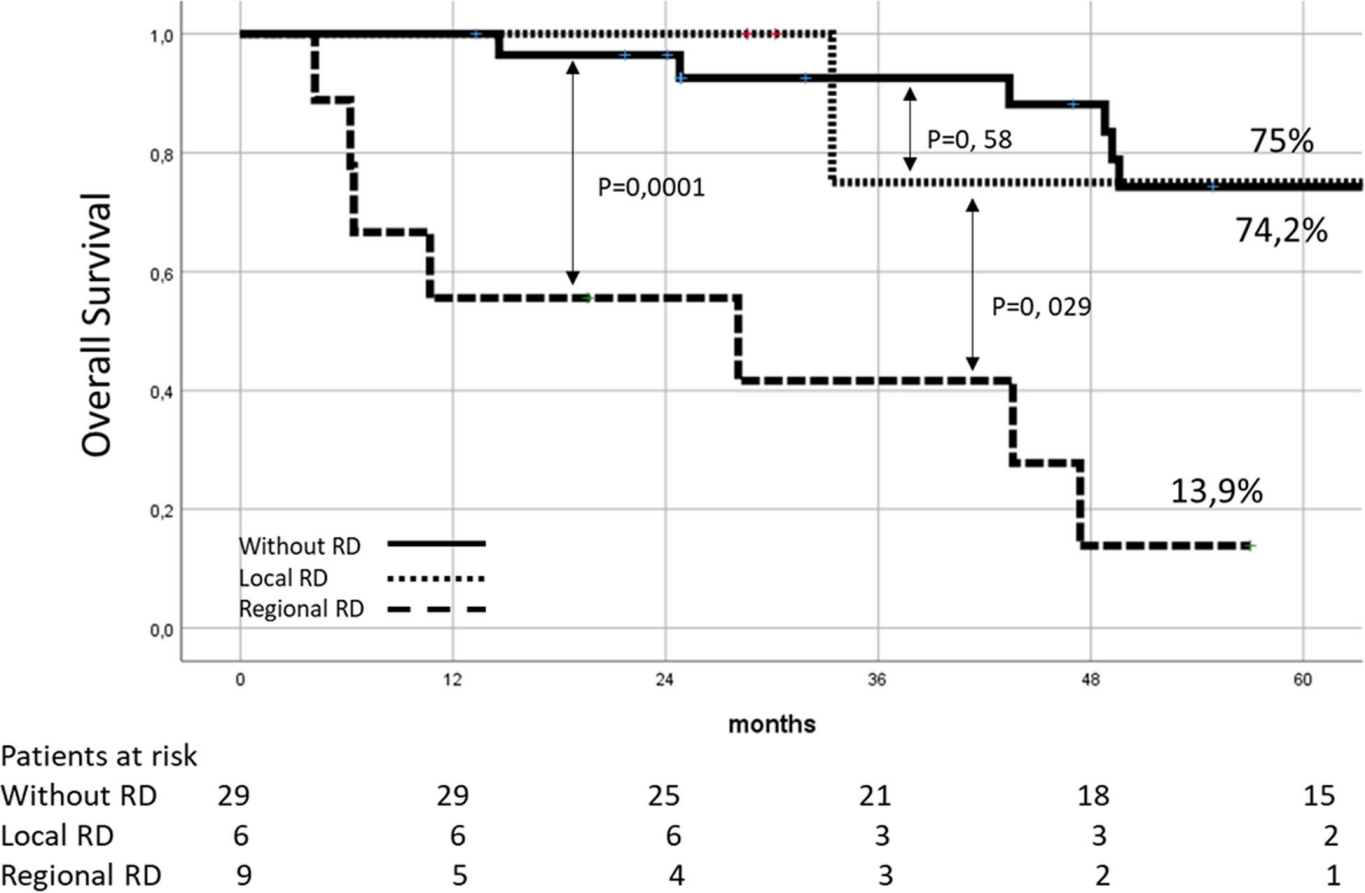
Long-term survival after an R0 resection (*n* = 44) for patients without RD (*n* = 29), with local RD (*n* = 6) and with regional RD (*n* = 9)

After selecting patients in which R0 resection was achieved (*n* = 44), 5-year survival rate for patients without RD (*n* = 29), with local RD (*n* = 6), and with regional RD (*n* = 9) was, respectively, 74.2%, 75%, and 13.9% (*p* = 0.0001) (Fig. 1).

### Predictive factors of regional and/or distant RD

Given that the survival of patients with local RD was similar to that of patients without RD, an analysis was performed to identify the predictive variables of regional and/or distant RD (RDRD) whose presence was associated with lower survival. In the univariate logistic regression analysis, T-stage, LVI, and PNI showed a statistically significant association with the presence of RDRD (Table 2).

**Table 2:** Analysis of predictive factors of regional and/or distant RD (RDRD). Fisher’s exact test and univariate logistic regression. Patients with unknown data had no significant differences regarding the risk of RDRD, compared to patients in the reference categories. This rules out bias due to missing values

	% Regional and/or distant residual disease	*P*	RR (CI 95%)	*P*
Cholecystectomy		0.975		0.975
Urgent	7/16 (43)		1.02 (0.37–3.38)	
Elective	17/43 (39.5)		REF	
Surgical approach		0.138		0.142
Open or converted	10/19 (52.6)		2.03 (0.755–7.05)	
Laparoscopic	13/40 (32.5)		REF	
Cystic margin invasion		0.658		0.660
Yes	7/16 (43.8)		1.10 (0.25–4.79)	
Unknown	6/13 (46.2)		0.64 (0.18–2.23)	
No	10/30 (33.3)		REF	
T-stage		**0.003**		**0.021**
T3	7/8 (87.5)		13.12 (1.48–116.26)	
T2	16/46 (34.8)		REF	
Tis + T1a + T1b	0/5 (0)		–	
Positive lymph nodes		0.412		0.420
N+	8/15 (53.3)		2.00 (0.40–9.83)	
Unknown	11/33 (33.3)		0.87 (0.21–3.64)	
N0	4/15 (36.4)		REF	
Grade		0.691		0.698
Poorly	2/7 (28.6)		1.06 (0.12–8.79)	
Moderately	11/24 (45.8)		2.25 (0.47–10.64)	
Unknown	7/17 (41.2)		1.86 (0.36–9.63)	
Well	3/11 (27.3)		REF	
Histology		0.241		0.247
Other	6/11 (54.5)		2.18 (0.58–8.24)	
Adenocarcinoma	17/38 (35.4)		REF	
Lymphovascular invasion		**0.005**		**0.010**
Yes	11/15 (73.3)		6.05 (1.54–23.73)	
Unknown	2/12 (16.7)		0.44 (0.81–2.39)	
No	10/32 (31.3)		REF	
Perineural invasion		**0.016**		**0.024**
Yes	11/16 (68.8)		6.16 (1.41–26.75)	
Unknown	7/24 (29.2)		1.15 (0.29–4.3)	
No	5/19 (26.3)		REF	

Significant data are in bold

The GBRS could be calculated in 25 patients (42.3%). No significant differences for any of the clinical, pathological, and operative variables included in Table 1, were observed compared with the rest of the patients included in the study thus ruling out a systematic bias.

In the group of patients with complete data available, the GBRS was the only predictive variable predictive of RDRD (*p* = 0.041) (Table 3). Values ranged from 5 to 9 (mean 6.48 ± 1.29). Twenty cases were in the intermediate risk category and only five in the high-risk category. The incidence of RDRD was 30% in patients with intermediate risk, and 80% in the high-risk category (*p* = 0.041). T2 stage patients represented the largest subgroup (*n* = 21), 17 belonged to the intermediate category and 4 to the high risk. The incidence of RDRD was 29.4% and 75% (*p* = 0.091), respectively. Three of the four T2 high-risk patients presented distant RD, compared to none in the intermediate risk group.

**Table 3:** Analysis of predictive histological factors of regional and/or distant residual disease (RDRD) in the 25 patients in which the GBRS could be calculated

	% Regional and/or distant residual disease	*P*
T-stage		0.113
T3	2/2 (100)	
T2	8/21 (38.1)	
Tis + T1a + T1b	0/2 (0)	
Grade		0.396
Poorly	2/4 (50)	
Moderately	6/12 (50)	
Well	2/9 (22.2)	
Lymphovascular invasion		0.126
Yes	4/6 (66.7)	
No	6/19 (31.6)	
Perineural invasion		0.096
Yes	6/10 (60)	
No	4/15 (26.7)	
GBRS		**0.041**
High	4/5 (80)	
Intermediate	6/20 (30)	
Low	0	

Significant data are in bold

## Discussion

Several studies have shown that RD represents a negative prognostic factor for survival after re-resection of IGBC [3, 7, 12, 16]. Results observed in our series are similar, but contrary to other published experiences, the prognostic value of RD depended on its anatomical situation.

Patients with local RD showed a 5-year survival rate similar to that of patients without RD (Fig. 1). In other studies [5, 10, 12, 17], local RD was associated with worse survival. This divergence in results could be due to the way in which liver involvement is classified (local extension of the tumor not excised in the first surgery vs metastatic disease). Continuity of the tumor with the gallbladder bed was observed in all our patients with isolated hepatic RD treated with an R0 resection. It was therefore a true local disease.

It has been suggested that the worsening of the prognosis related to RD could be due to disruption of the natural barriers between the tumor and the lymphatic network in the gallbladder bed or in the serosa layer, or to an intraoperative gallbladder perforation [18, 19].

The literature has highlighted the need to achieve an R0 resection with the aim of improving the prognosis of patients with IGBC [1, 4, 20]. However, in our study, the survival of patients with regional RD was similar to that of patients with distant RD, even after an R0 resection. The favorable results of the R0 resections observed in some publications are probably due to the inclusion in the analysis of patients without RD. This observation may support the administration of preoperative chemotherapy to patients with suspected regional RD. Unfortunately, imaging tests are not very reliable for the preoperative detection of RD. This could be explained by the small size of the tumoral disease that in many cases may be microscopic. It has been suggested that delaying preoperative staging up to 3 months after cholecystectomy may improve their results [15].

Given the limitations of radiological staging, attempts have been made to establish the risk of RD from histological findings in the cholecystectomy specimen [7, 16].

Similar to other studies [1–3, 7, 8, 12], T-stage in our series was a significant prognostic factor of RD. The prognostic value of other histological variables is difficult to assess due to the high proportion of missing data in most published series [7, 12, 16, 21].

In our series, besides T-stage, LVI and PNI were predictive factors of RDRD (Table 2). Together with the grade of differentiation, these are the factors used by Ethun et al. to develop the GBRS. The lack of significance of grade of differentiation in our study could be explained by the low proportion of patients with poorly differentiated tumors (12%). The incidence was 21% in the Ethun et al. study [16] and 40% in Creasy’s [7]. These data suggest relevant differences in patients’ characteristics among published series, which along with the problem of missing data can make it more difficult to obtain a useful score.

The GBRS could be calculated in 25 of the 59 patients (42.3%) and in this subgroup was the only predictive variable of RDRD (Table 3). The incidence of RDRD was significantly higher in high-risk patients than in intermediate-risk patients (80% vs 30%, *p* = 0.041). In T2-stage patients, the GBRS distinguished two groups with a difference in the incidence of RDRD, close to statistical significance (75% vs. 29.4%, *p* = 0.091). This observation may be especially relevant for clinical practice, since T2-stage [22] is the most frequent.

The recent reviews published by Søreide et al. [23] and Cherkassy et al. [24] emphasize the importance of tumor biology in the prognosis of patients with IGBC, as well as the need to select the patients who will undergo re-resection. In addition Cherkassky et al. propose a chemotherapy first approach in patients at high risk of micrometastatic disease. Therefore patients classified as high risk of RDRD by the GBRS could be eligible for this approach. In our series, three of the four T2-stage high-risk patients presented distant RD. However, this strategy should be indicated selectively, due to the risk of local progression or deterioration of functional status that could ultimately prevent re-resection.

Like other malignancies IGBC is a heterogeneous disease with various clinical and pathological presentations [25, 26]. In this scenario, a multiparameter score like GBRS could be a useful tool to complete a better prognostic assessment.

The present study has some obvious limitations due to its retrospective nature and the percentage of incomplete pathology data in the cholecystectomy specimen. However, data was prospectively collected and because of the statistical methodology used, it does not seem likely that the missing data could have introduced a significant bias in the results. However, given the sample size, the results should be considered with caution while waiting for new prospective studies to confirm the conclusions.

## Conclusions

Regional RD, but not local RD, in patients with IGBC constitutes a significant poor prognostic factor, even after an R0 resection. In our study, GBRS has been shown to be effective in identifying patients at high risk of RDRD.

## Data Availability

The datasets of the study are not publicly available due to legal restrictions but are available from the corresponding author on reasonable request.

## Abbreviations

CHT: Chemotherapy
GBRS: Gallbladder Cancer Risk Score
IGBC: Incidental gallbladder carcinoma
LVI: Lymphovascular invasion
OS: Overall survival
PNI: Perineural invasion
RD: Residual disease
RDRD: Regional and/or distant residual disease
